# Anti PD-1 treatment increases [^18^F]FDG uptake by cancer cells in a mouse B16F10 melanoma model

**DOI:** 10.1186/s13550-018-0433-1

**Published:** 2018-08-16

**Authors:** Mayu Tomita, Hironobu Yasui, Kei Higashikawa, Kohei Nakajima, Hideo Takakura, Tohru Shiga, Yuji Kuge, Mikako Ogawa

**Affiliations:** 1 Laboratory of Bioanalysis and Molecular Imaging, Graduate School of Pharmaceutical Sciences, Hokkaido University, Sapporo, Hokkaido 060-0812 Japan; 2 Central Institute of Isotope Science, Hokkaido University, Sapporo, Japan; 3 Graduate School of Medicine, Hokkaido University, Sapporo, Japan

**Keywords:** PD-1, Immune checkpoint inhibitor, [^18^F]FDG, Tumor microenvironment, Mouse melanoma

## Abstract

**Background:**

Programmed cell death 1 (PD-1) inhibitors act as immune checkpoint inhibitors and are more effective for improving survival time with less toxicity as compared with conventional chemotherapies. In anti PD-1 therapy, it is important to evaluate metabolism in the cancer microenvironment, as this helps to clarify the pathological conditions. Herein, we investigate the early effects of PD-1 therapy on 2-deoxy-2-[^18^F]fluoro-d-glucose ([^18^F]FDG) uptake in vivo, focusing on cell distribution and glycolysis in both cancer and immune cells.

**Results:**

In a B16F10 melanoma model, [^18^F]FDG-positron emission tomography (PET) was performed before treatment and 7 days after the start of treatment. Values were calculated as the percentage-injected activity per gram of tissue (%IA/g). Flow-cytometry was then performed to assess immune cell populations and glucose metabolism. There was a negligible difference in [^18^F]FDG uptake between tumors in the treatment group and non-treatment group before the treatment. In contrast, mean [^18^F]FDG uptake in the treatment group tumors was significantly higher (8.06 ± 0.48 %IA/g; *P* = 0.0074) than that in the non-treatment group (4.02 ± 1.03 %IA/g) after anti PD-1 treatment. Assessment of tumor immune cell populations showed that treatment slightly enriched CD8^+^ T cells and CD4^+^ T cells; however, infiltration of immune cells was negligible, and thus, immune cells were not responsible for the increase in [^18^F]FDG uptake. On the other hand, anti PD-1 treatment significantly increased glucose transporter 1 (GLUT1) and hexokinase II expression in CD45^−^ cancer cells, indicating that anti PD-1 treatment increased glucose metabolism in cancer cells.

**Conclusion:**

The present study shows that anti PD-1 therapy increases glucose metabolism in cancer cells.

**Electronic supplementary material:**

The online version of this article (10.1186/s13550-018-0433-1) contains supplementary material, which is available to authorized users.

## Background

The interaction between programmed cell death 1 (PD-1) and its ligand 1 (PD-L1) is a mechanism involved in the ability of immunogenic tumors to escape the immune response. Blockade of this interaction activates autoimmunity towards the tumor and provides a strategy for tumor immunotherapy [1]. Many classes of immune checkpoint inhibitors have been or are being developed, such as the PD-1 inhibitors (e.g., nivolumab) [2]. The results of clinical studies have shown that PD-1 inhibitors are more effective in improving survival time and less toxic than conventional chemotherapies [3], which has sparked worldwide interest in their therapeutic potential.

Immune checkpoint inhibitors target immune systems; thus, interaction between immune and tumor cells is important for therapeutic effects. Unlike in the case of chemotherapies which directly target cancer cells, immunotherapeutic effects appear late and sometimes after an initial increase in tumor burden or the appearance of new lesions [4]. Therefore, therapeutic effects in the early stages of treatment need to be determined not by tumor volume. Thus, molecular imaging technique (e.g., positron emission tomography; PET) should be helpful for evaluating the therapeutic effect.

Along with the development of immune checkpoint inhibitors, metabolism in the cancer microenvironment has received a great deal of attention because it is important for understanding how tumor and immune cells share or compete for resources and how such relationships regulate antitumor immunity [4, 5]. For example, it has been proposed that T cell activation requires metabolic reprogramming including that of glycolysis [6]. However, recent studies have demonstrated that the glycolytic activity of cancer cells may restrict glucose utilization by tumor-infiltration lymphocytes (TILs), thereby impairing antitumor immunity, and that checkpoint blockade antibodies restore glucose in the tumor microenvironment, permitting T cell glycolysis and interferon-gamma (IFN-*γ*) production [7, 8]. In this way, immune checkpoint blockade alters the tumor microenvironment and causes immune cell infiltration and activation. Thus, tumor uptake of 2-deoxy-2-[^18^F]fluoro-d-glucose ([^18^F]FDG) should be increased due to activation of these cells. Some clinical studies have in fact shown that immune checkpoint inhibitors increase [^18^F]FDG uptake [9, 10]; however, others have shown the opposite effect [9, 11, 12]. Anti PD-1 and anti-cytotoxic T-lymphocyte-associated antigen 4 (CTLA-4) antibodies are used as checkpoint inhibitors in clinic. These are different molecule-targeted immuno-therapeutics, and the response pattern might be different. In fact, there is not much known about the early response pattern of PD-1-therapy in [^18^F]FDG-PET and most of the study deal with CTLA-4.

These unidentified points about [^18^F]FDG uptake in anti PD-1 therapy may be described by focusing on cancer cells, which are also known as high-glycolysis cells [13]. However, few studies to date have focused on cancer cell metabolism in immune checkpoint therapy. It is important to evaluate metabolism not only in infiltrated immune cells but also of cancer cells in the cancer microenvironment, as doing so can contribute to clarifying pathological conditions and also has applications for imaging. Herein, we report on the early effect of PD-1 therapy on [^18^F]FDG uptake in vivo, focusing on cell distribution and glycolysis in both cancer and immune cells.

## Methods

### Cell line

The B16F10 melanoma cell line was purchased from ATCC (Manassas, VA, USA) in 2016. Cells were cultured in Dulbecco’s modified Eagle medium supplemented with 10% fetal bovine serum (FBS), 1% penicillin-streptomycin, and 2 mmol/L l-glutamine.

### Mouse models

**Fig. 1:**
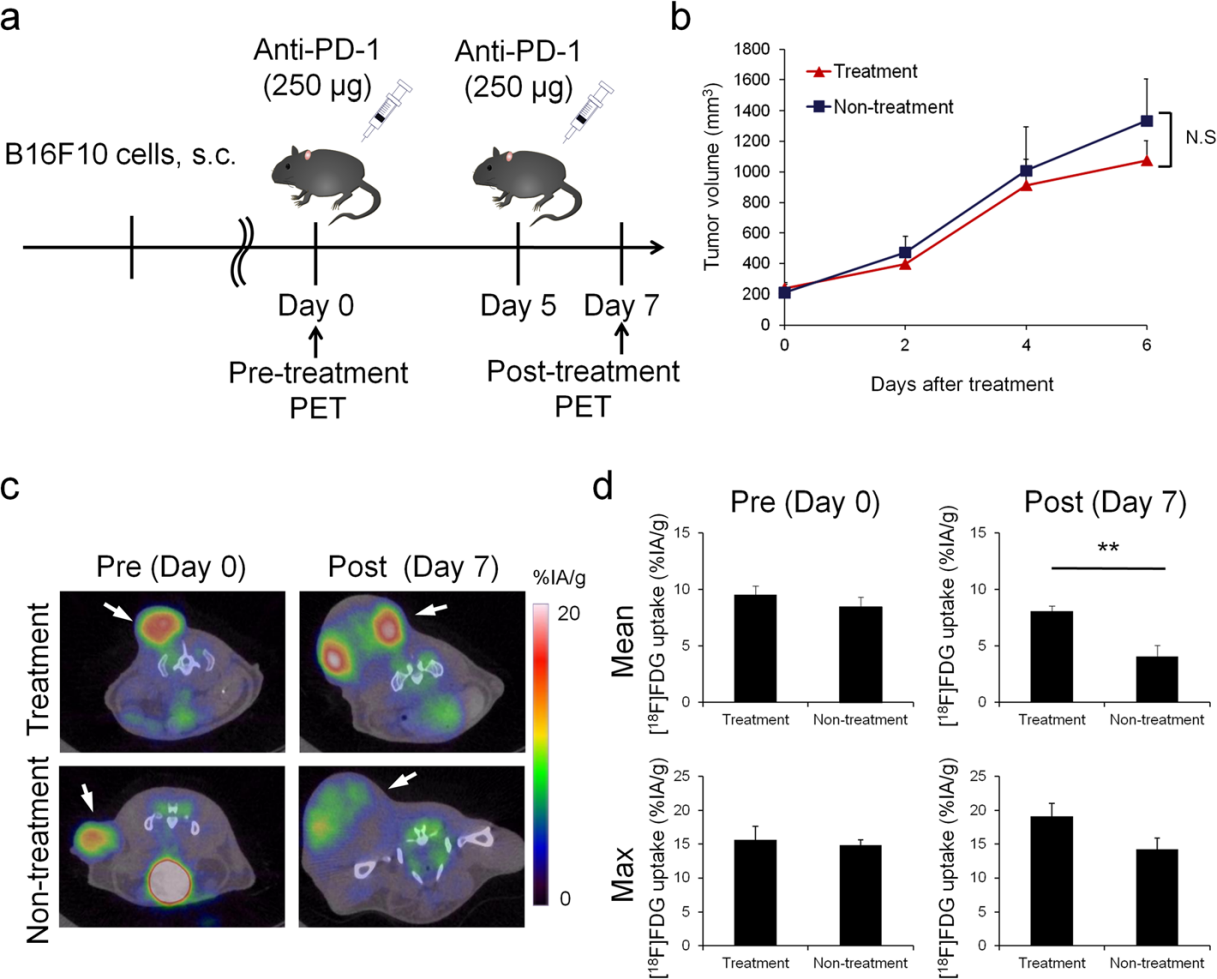
**a** Timeline diagrams for animal studies. **b** In vivo B16F10 tumor growth curves after anti-PD-1 treatment (*n* = 25–27). **c** Coronal sections from [^18^F]FDG-PET/CT imaging performed on a treated (top) or non-treated (bottom) mouse on day 0 and day 7. **d** Mean (top) and maximum (bottom) [^18^F]FDG uptake values in tumor on day 0 and day 7 (*n* = 5). Data represent mean ± SEM; **P < 0.01; N.S., not significant

Animal care, experiments, and euthanasia were performed in accordance with protocols approved by the Hokkaido University Animal Research Committee. Male C57BL/6JJmsSlc mice (7–10 weeks old) were purchased from Sankyo Labo Service Corporation, Inc. (Tokyo, Japan). Animal experiments were performed in specific pathogen-free facilities. Mice were inoculated subcutaneously with 5 × 10^5^ B16F10 cells in 100 μL of phosphate-buffered saline (PBS). Anti PD-1 treatment was started when tumor presence was confirmed. Anti-mouse PD-1 (clone RMP1-14) was purchased from BioXcell (West Lebanon, NH, USA). Anti PD-1 antibody (250 μg in 200 μL PBS) was administrated twice i.p. 5 days apart. Tumor measurements were made two to three times weekly using calipers, and the volume was expressed in mm^3^ [0.5 × L × W^2^] (L, long diameters; W, short diameters of the tumor). [^18^F]FDG-PET/Computed Tomography (CT) imaging was performed just prior to initiating therapy and at 7 days after the initiation of anti PD-1 treatment initiation (Fig. 1a). The end-point was when tumor size reached 10% of the body weight.

In this study, treatment group was defined as the group which was treated by anti PD-1 antibody twice a week and non-treatment group was defined as the group which was not treated by anti PD-1 antibody. Fifty-seven mice were used in total (treatment group *n* = 27, non-treatment group *n* = 25, pre-treatment group *n* = 5); among these mice, 10 mice for PET (treatment group *n* = 5, non-treatment group *n* = 5), 40 mice for flow-cytometry (treatment group *n* = 18, non-treatment group *n* = 17, pre-treatment group *n* = 5, 2–4 markers were analyzed in each mouse, and *n* = 5–6 for each marker), and 4 mice for histopathology and autoradiography (treatment group *n* = 2, non-treatment group *n* = 2).

### PET imaging

C57/BL6 mice were fasted for 12 h immediately prior to injection of [^18^F]FDG. [^18^F]FDG (3.5 MBq) in 100 μL of saline was administrated to mice via a lateral tail vein. [^18^F]FDG-PET/CT images were acquired on an Inveon small-animal multimodality PET/CT system (Siemens Medical Solutions, Knoxville, TN, USA) [14]. During imaging, anesthesia was maintained with 1% isoflurane. CT scanning was performed from 20 min after the tracer injection, and PET scanning was performed for 10 min beginning 40 min after the tracer injection.

Acquired PET-CT images were reconstructed using the filtered back projection (FBP) algorithm with the ramp filter cut-off at the Nyquist frequency. The image matrix was 256 × 256 × 318, resulting in a voxel size of 0.388 × 0.388 × 0.398 mm^3^. CT was performed for attenuation correction. The half rotation step-and-shoot mode was used for CT data acquisition. The radiographic source was operated at 500 μA and 80 kVp. Images were reconstructed using a Feldkamp cone-beam algorithm, with a Shepp-Logan filter.

PET-CT images and three-dimensional regions of interest (volume of interest; VOI) of tumors were computed using Inveon Research Workplace software (Siemens Medical Solutions). All radioactivity concentration values were normalized according to the percentage-injected activity per gram of tissue (%IA/g), and the maximum or mean %IA/g value obtained in VOI was considered for quantitative analysis.

After PET imaging on day 7, the mice were sacrificed and their organs were dissected. Tissues (tumor xenografts, spleens, and blood) were weighed, and radioactivity was measured using a gamma counter (2480 Wizard 2 gamma counter, PerkinElmer, Waltham, MA, USA). Data were calculated as %IA/g.

### Flow-cytometry analysis

Tumors were harvested and processed using Collagenase I and DNase I (Wako, Osaka, Japan). The resulting cell suspensions were clarified using 40-μm filters to prepare single cell suspensions, and single cells were suspended in PBS supplemented with 2% FBS. Splenocytes were hemolyzed and incubated with anti-CD16/32 2.4G2 (BD Biosciences, San Jose, CA, USA) to reduce FcγR binding. Cell-surface antigens were stained with antibodies specific for CD8 (BioLegend, San Diego, CA, USA, clone 53-6.7), CD4 (BioLegend, clone GK1.5), CD45 (BioLegend, clone 30-F11), IA/IE (BD Bioscience, clone M5/114), CD11c (BD Bioscience, clone HL3), CD11b (BioLegend, clone M1/70), and F4/80 (BioLegend, clone BM8).

For intracellular staining, cells were fixed and permeabilized using Foxp3/Transcription Factor Staining Buffer Set (eBioscience, San Diego, CA, USA) after cell surface staining and were stained with labeled antibodies against the intracellular molecules foxp3 (eBioscience, clone FJK-16s), glucose transporter 1 (GLUT1, Abcam, Cambridge, UK, clone EPR3915), and hexokinase II (HX2, Abcam, clone EPR20839).

Samples were analyzed on a FACS Calibur HG flow cytometer (BD Biosciences). Data analysis was performed with CellQuest™ software (Becton Dickinson, Lincoln Park, NJ, USA).

### Histopathology and autoradiography

C57/BL6 mice (*n* = 2) were fasted for 12 h, and then [^18^F]FDG (3.5 MBq) was injected via the tail vein. The mice were sacrificed 40 min after the [^18^F]FDG injection, and tumor tissues were embedded in optimal cutting temperature (OCT) compound and immediately snap-frozen. Serial 5-μm tumor sections were placed onto glass slides, dried, and fixed at room temperature. Slices were used for autoradiography and hematoxylin-eosin (HE) staining. Autoradiograms were obtained using a phosphor imaging system (FLA-7000, Fujifilm, Tokyo, Japan).

### Statistics

Statistical analyses were performed using JMP pro 3.0 software (SAS Institute Inc., Cary, NC, USA). Results are expressed as mean ± SEM. Statistical significance was determined by two-tailed Student’s or Welch’s *t* test, and a *P* value less than 0.05 was considered statistically significant.

## Results

### In vivo [^18^F]FDG-PET monitoring for the response to anti PD-1 treatment

Anti PD-1 treatment did not significantly inhibit tumor growth (Fig. 1b). PET-CT scans and mean/maximum [^18^F]FDG uptake values in tumors are shown in Fig. 1c, d. There was a minimal difference in [^18^F]FDG uptake between tumors in the treatment group and non-treatment group before treatment. In contrast, the mean uptake of [^18^F]FDG in treatment group tumors was significantly higher (8.06 ± 0.48 %IA/g; *P* = 0.0074) than that in non-treatment group tumors (4.02 ± 1.03 %IA/g) after anti PD-1 treatment. Furthermore, the maximum uptake of [^18^F]FDG in the treatment group tumors tended to be higher (19.14 ± 1.86 %IA/g; *P* = 0.0839) than that in the non-treatment group (14.24 ± 1.64 %IA/g).

### Ex vivo validation

**Fig. 2:**
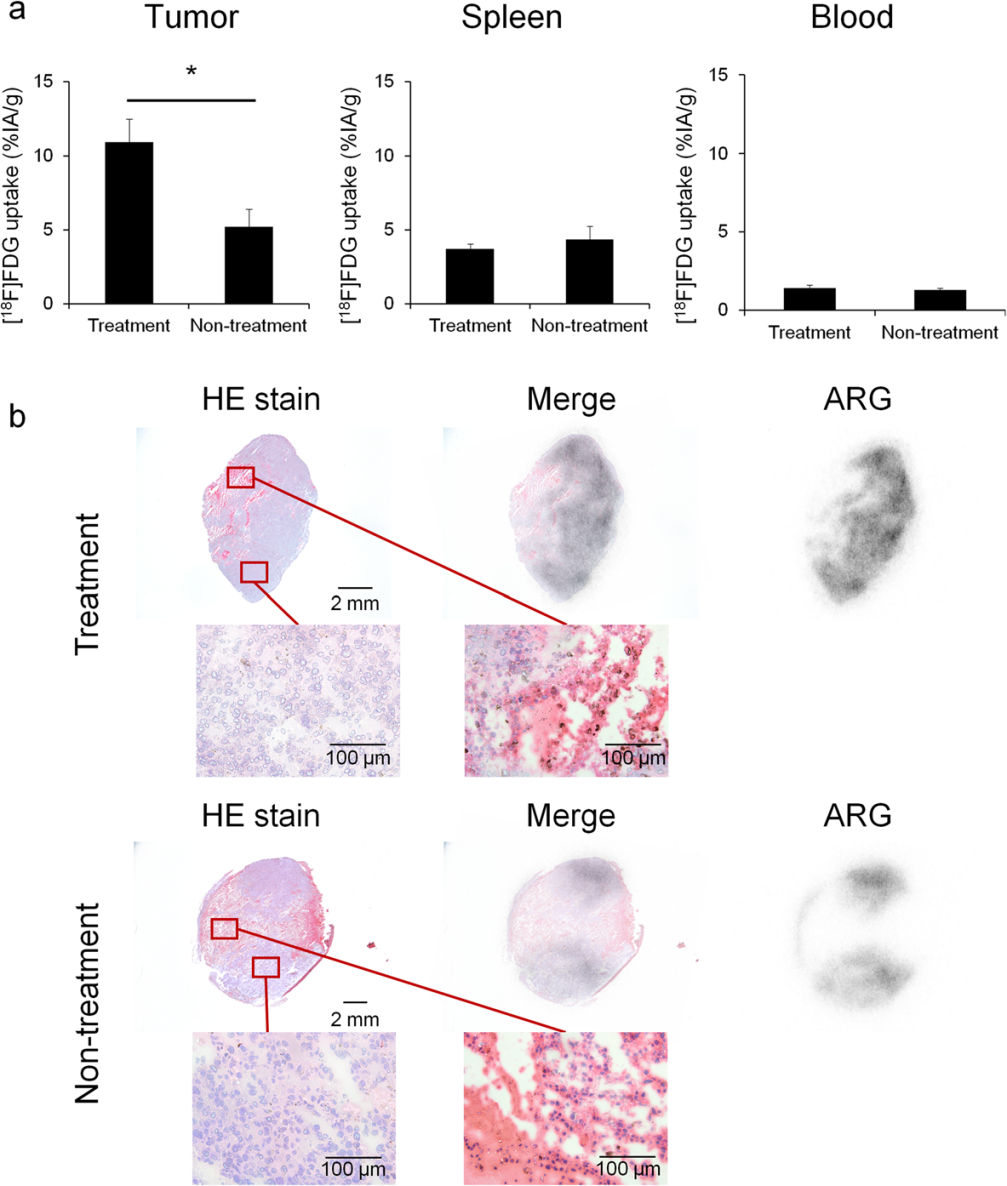
Ex vivo validation of [^18^F]FDG uptake. **a** Evaluated the percentage-injected activity per gram of tissue (%IA/g) by gamma counter in tumors, spleens and blood on day 7 (*n* = 7). **b** Representative HE-stained and autoradiography (ARG) images of treatment group tumor (top) or non-treatment group tumor (bottom). Data represent mean ± SEM; *P < 0.05

As was the case with PET-CT, the uptake of [^18^F]FDG in treatment group tumors was significantly different from that of the non-treatment group (*P* = 0.0126), but the uptake of [^18^F]FDG in the spleen and blood did not differ from that of the non-treatment group (Fig. 2a).

The regional distribution of [^18^F]FDG was assessed by autoradiography, and the autoradiographs were compared with HE-stained samples (Fig. 2b). The results confirmed the uptake of [^18^F]FDG in non-necrotic areas, and the pathological images showed no significant difference between the treatment group and the non-treatment group.

### Analysis of immune cell population in tumor and spleen

**Fig. 3:**
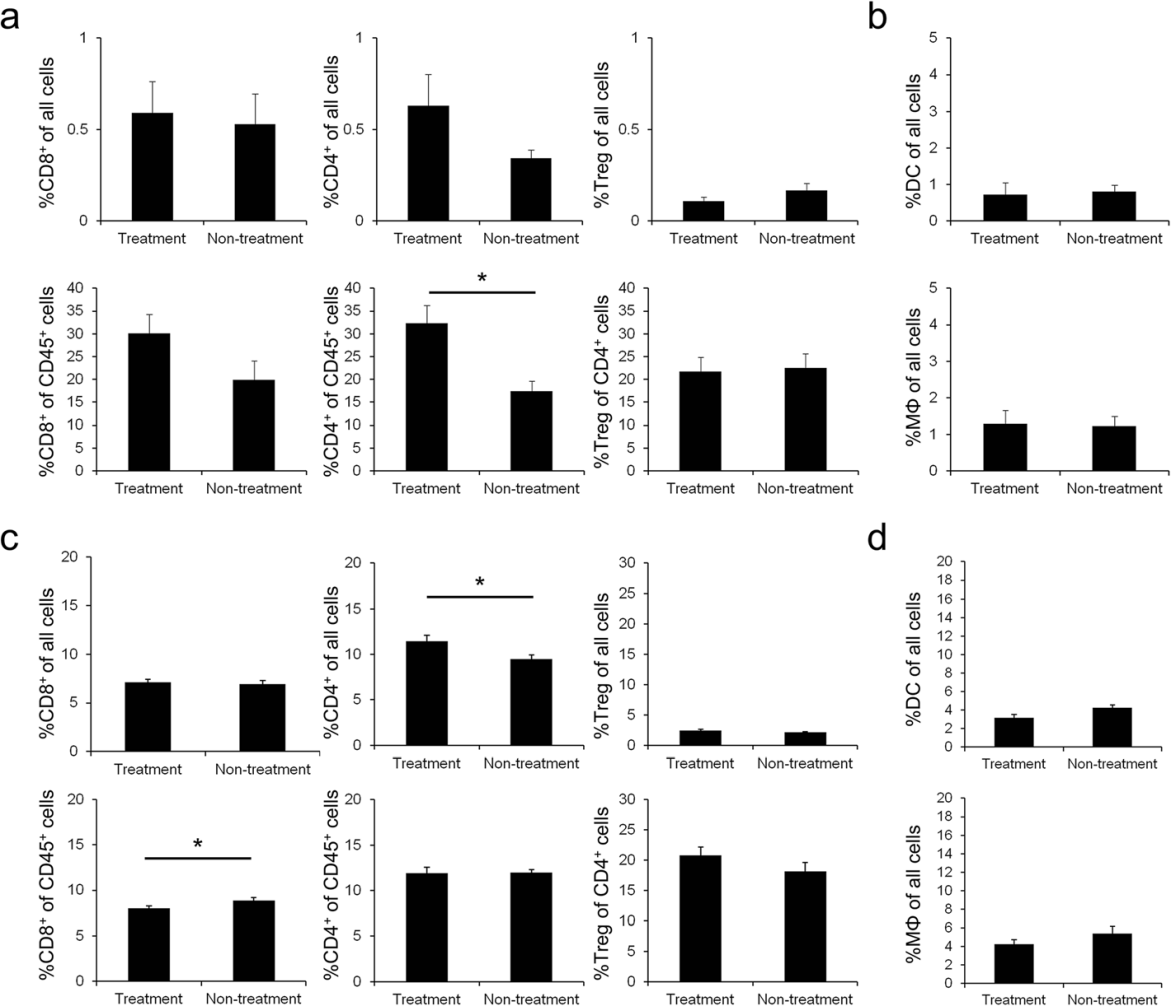
Flow-cytometry analysis of immune cell populations. **a** Flow-cytometry analysis of CD8^+^ cells (left), CD4^+^ cells (middle), and Foxp3^+^ cells (Treg, right) of all cells or gated cells in tumor on day 7 (*n* = 6). **b** Flow-cytometry analysis of IA/IE^+^ CD11c^+^ cells (DC, top) and F4/80^+^ CD11b^+^ cells (MΦ, bottom) of all cells in tumor on day 7 (*n* = 6). **c** Flow-cytometry analysis of CD8^+^ cells (left), CD4^+^ cells (middle), and Foxp3^+^ cells (right) of all cells or gated cells in spleen on day 7 (*n* = 6). **d** Flow-cytometry analysis of IA/IE^+^ CD11c^+^ cells (top) and F4/80^+^ CD11b^+^ cells (bottom) of all cells in spleen on day 7 (*n* = 6). Data represent mean ± SEM; *P < 0.05

Flow-cytometry was performed to assess tumor and spleen immune cell populations in this tumor model, and the effect of immune cells on [^18^F]FDG uptake was analyzed. Anti PD-1 treatment increased CD8^+^ and CD4^+^ T cells, although the difference was not significant except for %CD4^+^ of CD45^+^ cells (Fig. 3a). There was no effect on Treg infiltration. But since the infiltration levels of these cells were small, the results should not largely affect [^18^F]FDG uptake. Among CD45^+^ tumor cells, anti PD-1 treatment significantly increased the frequency of effector CD4^+^ T cells (Fig. 3a). In addition, among all tumor cells, anti PD-1 treatment did not lead to increased frequencies of F4/80^+^ CD11b^+^ macrophages (MΦ) and IA/IE^+^ CD11c^+^ dendritic cells (DC) (Fig. 3b). Infiltration of these immune cells (T cells, MΦ and DC) into tumor tissues was negligible at around 1%.

In contrast, anti PD-1 treatment significantly enriched CD4^+^ T cells among CD45^+^ splenic cells and significantly reduced CD8^+^ T cells among all splenic cells (Fig. 3c). However, the change in the number of splenic T cells was only around 2% and it should not affect the [^18^F]FDG uptake in the spleen. Additionally, anti PD-1 treatment did not affect the ratio of MΦ and DC to all splenic cells (Fig. 3d).

### Tumor and splenic metabolism of glucose

We measured the expression of GLUT1 and hexokinase II in tumors to better understand glucose metabolism in cancer and immune cells.

**Fig. 4:**
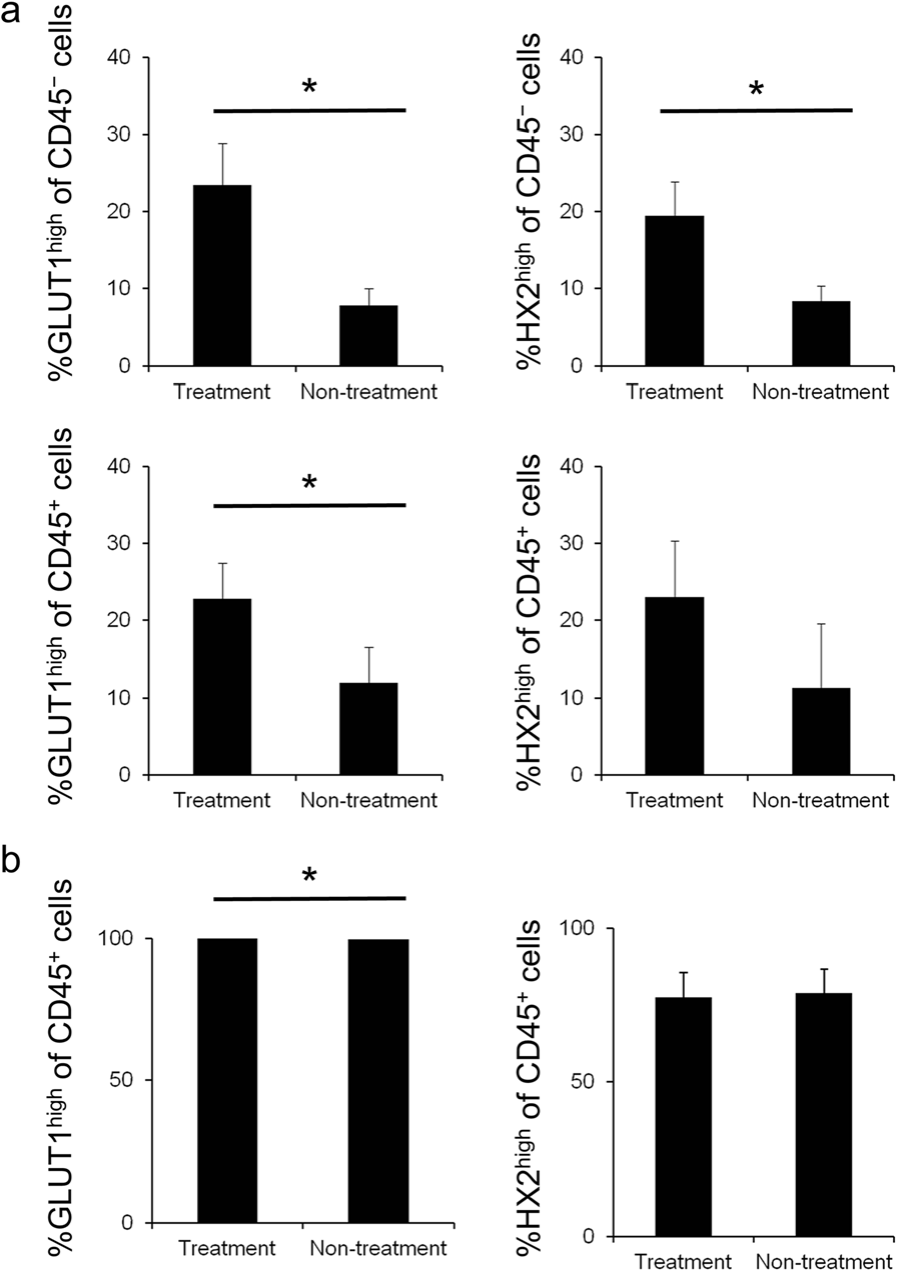
Flow-cytometry analysis of metabolism of glucose. **a** Flow-cytometry analysis of high expression cells of glucose metabolism markers; GLUT1 (left) and hexokinase II (right). Values represent percentage of CD45^−^ cancer cells (top) or CD45^+^ immune cells (bottom) in tumor on day 7 (*n* = 6). **b** Flow-cytometry analysis of high expression cells of glucose metabolism markers; GLUT1 (left) and hexokinase II (right) of CD45^+^ immune cells in spleen on day 7 (*n* = 6). Data represent mean ± SEM; *P < 0.05

Figure 4a shows GLUT1^high^ cells/hexokinase II^high^ cells of CD45^−^ cells or CD45^+^ cells in tumors. Anti PD-1 treatment significantly increased GLUT1^high^ cells and hexokinase II^high^ cells of CD45^−^ cells, which were mostly cancer cells (*P* = 0.0251 and *P* = 0.0467, respectively). Furthermore, anti PD-1 treatment significantly increased GLUT1^high^ cells among CD45^+^ immune cells (*P* = 0.0390). Additional file 1: Figure S1 indicates that baseline of GLUT1 and hexokinase II expression in cancer cells on day 0 was in the same level as it in the treatment group on day 7. This result is consistent with mean [^18^F]FDG uptake values on day 0 and day 7 shown by PET-CT.

Anti PD-1 treatment significantly increased percentage of GLUT1^high^ cells in CD45^+^ immune cells in the spleen (*P* = 0.045, Additional file 1: Table S1). However, the difference was less than 0.5% and does not affect the [^18^F]FDG results (Fig. 4b).

## Discussion

[^18^F]FDG is a widely used radiotracer for cancer imaging. Tumor cells and inflammatory immune cells (e.g., T cells and MΦ activated in an inflammatory setting) exhibit accelerated glycolysis [4], and [^18^F]FDG can be used to diagnose cancer and immune diseases using this metabolic characteristic [15]. [^18^F]FDG uptake in tumors treated by anti PD-1 therapy may differ from that in tumors treated by conventional chemotherapies because immune checkpoint blockade changes the tumor microenvironment including that of tumor cells and immune cells. Given the uncertainties regarding [^18^F]FDG uptake in anti PD-1 therapy, it is important to clarify exactly how PD-1 therapy affects [^18^F]FDG uptake by focusing on cell distribution and glycolysis in both cancer and immune cells.

Thus, in this study, experiments were conducted using a B16F10 melanoma model, which is popular for PD-1 blockade studies, focusing on changes in immune cell distribution and the functional glucose metabolic rate. Significant inhibition of tumor growth was not detected by anti PD-1 treatment during our observation period (7 days), but it is reported that tumor growth would be inhibited 12 days after anti PD-1 treatment in the same animal model [16]. However, [^18^F]FDG uptake in the treatment group tumors was higher than that in the non-treatment group tumors following treatment, as shown by PET-CT and ex vivo validation, on day 7. Since the tumor growth was very fast in this animal model and necrotic lesion in the tumor was detected on day 7, decrease in [^18^F]FDG uptake was observed in non-treatment group. Other studies have reported the use of [^18^F]FDG-PET/CT scanning for predicting the early response to immune checkpoint inhibitors clinically [9–12]. One such study reported that [^18^F]FDG uptake in treatment-responsive patients increased in the early stage of immune checkpoint inhibition therapy for melanoma [10] which agrees with our results. This clinical study also suggests that increased [^18^F]FDG uptake may correlate with eventual clinical benefit. Our observation period in this mouse model should be too early to see the therapeutic response, since the model is poorly immunogenic. But, there is a report that significant therapy response could be observed even after the first cycle of anti PD-1 therapy [17]. This would also explain why there was no increased [^18^F]FDG-uptake in the spleen observed, although it is reported in other studies [18, 19].

As for increased [^18^F]FDG uptake in treatment group tumors, we assumed that infiltration of inflammatory immune cells caused [^18^F]FDG uptake to be increased. To clarify which immune cells contribute to increase [^18^F]FDG uptake and determine how they affect [^18^F]FDG uptake, flow-cytometry analysis was performed. The results of the assessment of tumor immune cell populations in tumors showed that anti PD-1 treatment slightly enriched CD8^+^ and CD4^+^ T cells, but it did not affect the infiltration of Tregs, MΦ, and DC. Not all kinds of immune cells were investigated in our study, and such investigation should be useful to further understand the observation in the future. Moreover, infiltration of these immune cells into tumor tissues was negligible so this result cannot explain the increase in [^18^F]FDG uptake. In fact, the B16 melanoma model is poorly immunogenic compared to the CT26 colon model [20]. In clinical studies, patients who respond to PD-1 blockade often show high numbers of CD8-expressing cells in tumors [21]. Therefore, in a strongly immunogenic mouse model, infiltration of inflammatory immune cells may cause [^18^F]FDG uptake to be increased.

In our B16F10 tumor model, we performed flow-cytometry analysis focusing on GLUT1 and hexokinase II to clarify the mechanism of increased [^18^F]FDG uptake. Anti PD-1 treatment significantly increased GLUT1 expression in CD45^+^ immune cells. But, immune cells did not contribute substantially to the increase in [^18^F]FDG uptake because infiltration was little. On the other hand, anti PD-1 treatment significantly increased GLUT1^high^ cells and hexokinase II^high^ cells among CD45^−^ cancer cells, which indicates that anti PD-1 treatment increased glucose metabolism in each cancer cell. Such increased glucose metabolism of each cancer cell might be affected by activated immune cells. For instance, inflammatory cytokine tumor necrosis factor α (TNFα) induces glycolysis in prostate epithelial cell models [22]. Also, there is a report that expression of PD-L1 is associated with the expression of GLUT1 [7, 23], and Haratake et al. have reported positive conversion of PD-L1 expression on cancer cells after anti PD-1 treatment [24].

Additional work is needed to identify the mechanisms by which glycolysis is increased in cancer cells, which may ultimately help to make PD-1 therapy more effective. [^18^F]FDG uptake in tumors might reflect the influence of necrosis. However, increase in GLUT1 (and hexokinase II) expression indicates that anti PD-1 treatment elevates glycolysis in cancer cells. Therefore, the increase in [^18^F]FDG uptake could be explained by the elevation of glycolysis in cancer cells. The increased glucose metabolism in cancer cells might sensitively reflect activation of tumor-infiltrated immune cells mediated by the therapy.

Notably, the increase in [^18^F]FDG uptake occurred only in tumors and not in spleen. Flow-cytometry analysis reveals that anti PD-1 treatment resulted in a minimal change in the composition of immune cells and a negligible effect on glucose metabolism by CD45^+^ T cells in the spleen. This agrees with our results that anti PD-1 treatment did not affect splenic [^18^F]FDG uptake. Other studies have reported that inflammatory diseases like malaria or postsurgical adjuvant melanoma therapy with high-dose interferon-α-2b increase [^18^F]FDG uptake in the spleen [25]. However, our results suggest that PD-1 blockade does not increase splenic [^18^F]FDG. Therefore, the effect of PD-1 blockade on [^18^F]FDG uptake in the spleen may differ from such inflammatory responses. This result supplemented the finding that increased [^18^F]FDG uptake in tumors was caused not by immune cells but rather by cancer cells.

PD-1 blockade represents a novel and paradigm-changing mechanism of action, and for refractory cancer typified by melanoma, immune checkpoint therapy is promising. However, PD-1 inhibitors are not effective in all patients [26–28]. [^18^F]FDG is a widely used PET tracer for cancer imaging, but the value of [^18^F]FDG for monitoring anti PD-1 therapy is unclear because the tumor microenvironment is changed by activated immune cells. Therefore, it is important to know how immune checkpoint therapy in cancer therapy affects [^18^F]FDG uptake. Our results suggest that anti PD-1 therapy increases [^18^F]FDG uptake by cancer cells, although this could be temporal. This information is helpful both for understanding the pathological conditions associated with anti PD-1 therapies and for application to imaging. Future studies could examine the value of measuring [^18^F]FDG uptake to monitor the therapeutic effects of anti PD-1 therapy; for example, by comparing responsive and non-responsive models with strict comparison of the time course of changes in [^18^F]FDG uptake and glucose metabolism in cancer cells, immune cells, and entire tumors. It is also needed more long-term experiment including in therapy-responded animal model.

## Conclusion

The present study is the first to assess initial changes in [^18^F]FDG uptake following anti PD-1 treatment in vivo. And this shows that anti PD-1 therapy increases glucose metabolism by cancer cells at the point when anti PD-1 therapy does not cause significant inhibition of tumor growth.

## Additional file

Additional file 1: **Figure S1.** Flow-cytometry analysis of metabolism of glucose. a Flow-cytometry analysis of high expression cells of glucose metabolism markers; GLUT1 and hexokinase II. Values represent percentage of CD45^−^ cancer cells (left) or CD45^+^ immune cells (right) in tumor on day 0 (*n* = 5). b Flow-cytometry analysis of high expression cells of glucose metabolism markers; GLUT1 and hexokinase II of CD45^+^ immune cells in spleen on day 0 (*n* = 5). Data represent mean ± SEM. **Table S1.** Complete data about flow-cytometry analysis of high expression cells of glucose metabolism markers; GLUT1 and hexokinase II of CD45^+^ immune cells in spleen on day 7. (PDF 194 kb)

## References

[1] Iwai Y, Ishida M, Tanaka Y, Okazaki T, Honjo T, Minato N (2002). Involvement of PD-L1 on tumor cells in the escape from host immune system and tumor immunotherapy by PD-L1 blockade. Proc Natl Acad Sci U S A.

[2] Okazaki T, Chikuma S, Iwai Y, Fagarasan S, Honjo T (2013). A rheostat for immune responses: the unique properties of PD-1 and their advantages for clinical application. Nat Immunol.

[3] Zhang T, Xie J, Arai S (2016). The efficacy and safety of anti-PD-1/PD-L1 antibodies for treatment of advanced or refractory cancers: a meta-analysis. Oncotarget.

[4] Andrejeva G, Rathmell JC (2017). Similarities and distinctions of cancer and immune metabolism in inflammation and tumors. Cell Metab.

[5] Buck MD, Sowell RT, Kaech SM, Pearce EL (2017). Metabolic instruction of immunity. Cell.

[6] Almeida L, Lochner M, Berod L, Sparwasser T (2016). Metabolic pathways in T cell activation and lineage differentiation. Semin Immunol.

[7] Chang CH, Qiu J, O’Sullivan D (2015). Metabolic competition in the tumor microenvironment is a driver of cancer progression. Cell.

[8] Ho PC, Bihuniak JD, MacIntyre AN (2015). Phosphoenolpyruvate is a metabolic checkpoint of anti-tumor T cell responses. Cell.

[9] Kong BY, Menzies AM, Saunders CAB (2016). Residual FDG-PET metabolic activity in metastatic melanoma patients with prolonged response to anti-PD-1 therapy. Pigment Cell Melanoma Res.

[10] Cho SY, Lipson EJ, Im H-J, et al. Prediction of response to immune checkpoint inhibitor therapy using early time-point FDG-PET/CT imaging in patients with advanced melanoma. J Nucl Med. 2017;58:1421–28. 10.2967/jnumed.116.188839 PMC5577627 28360208

[11] Sachpekidis C, Larribere L, Pan L, Haberkorn U, Dimitrakopoulou-Strauss A, Hassel JC (2015). Predictive value of early 18F-FDG PET/CT studies for treatment response evaluation to ipilimumab in metastatic melanoma: preliminary results of an ongoing study. Eur J Nucl Med Mol Imaging.

[12] Higuchi M, Owada Y, Inoue T (2016). FDG-PET in the evaluation of response to nivolumab in recurrent non-small-cell lung cancer. World J Surg Oncol.

[13] Liberti MV, Locasale JW. The Warburg effect: how does it benefit cancer cells? Trends Biochem Sci. 2016;41(3):211–218. 10.1016/j.tibs.2015.12.001 PMC4783224 26778478

[14] Magota K, Kubo N, Kuge Y, Nishijima KI, Zhao S, Tamaki N (2011). Performance characterization of the Inveon preclinical small-animal PET/SPECT/CT system for multimodality imaging. Eur J Nucl Med Mol Imaging.

[15] Vaidyanathan S, Patel CN, Scarsbrook AF, Chowdhury FU (2015). FDG PET/CT in infection and inflammation - current and emerging clinical applications. Clin Radiol.

[16] Chen S, Lee L-F, Fisher TS (2014). Combination of 4-1BB agonist and PD-1 antagonist promotes antitumor effector/memory CD8 T cells in a poorly immunogenic tumor model. Cancer Immunol Res.

[17] Seith F, Forschner A, Schmidt H, et al. 18F-FDG-PET detects complete response to PD1-therapy in melanoma patients two weeks after therapy start. Eur J Nucl Med Mol Imaging. 2018;45:95–101. 10.1007/s00259-017-3813-2 28831583

[18] Dercle L, Seban R, Lazarovici J, et al. PET and CT scans detect new imaging patterns of response and progression in patients with Hodgkin lymphoma treated by anti–programmed death 1 immune checkpoint inhibitor. J Nucl Med.2018;59:15–25. 10.2967/jnumed.117.193011 28596157

[19] Radu CG, Shu CJ, Nair-gill E, et al. Molecular imaging of lymphoid organs and immune activation by positron emission tomography with a new [18F]-labeled 2'-deoxycytidine analog. Nat Med. 2008;14:783–8. 10.1038/nm1724 PMC2720060 18542051

[20] Lechner MG, Karimi SS, Barry-Holson K (2013). Immunogenicity of murine solid tumor models as a defining feature of in vivo behavior and response to immunotherapy. J Immunother.

[21] Tumeh PC, Harview CL, Yearley JH (2014). PD-1 blockade induces responses by inhibiting adaptive immune resistance. Nature.

[22] Vaughan RA, Garcia-smith R, Trujillo KA, Bisof M. Tumor necrosis factor alpha increases aerobic glycolysis and reduces oxidative metabolism in prostate epithelial cells. Prostate. 2013;1546:1538–46. 10.1002/pros.22703 23818177

[23] Kasahara N, Kaira K, Bao P (2018). Lung cancer correlation of tumor-related immunity with 18F-FDG-PET in pulmonary squamous-cell carcinoma. Lung Cancer.

[24] Haratake N, Toyokawa G, Tagawa T (2017). Positive conversion of PD-L1 expression after treatments with chemotherapy and nivolumab. Anticancer Res.

[25] Liu Y (2009). Clinical significance of diffusely increased splenic uptake on FDG-PET. Nucl Med Commun.

[26] Callahan MK, Postow MA, Wolchok JD (2016). Targeting T cell co-receptors for cancer therapy. Immunity.

[27] Philips GK, Atkins M (2018). Therapeutic uses of anti-PD-1 and anti-PD-L1 antibodies. Int Immunol.

[28] Tumeh PC, Harview CL, Yearley JH (2015). PD-1 blockade induces responses by inhibiting adaptive immune resistance. Nature.

