# Supplementary material for: Anti PD-1 treatment increases [18F]FDG uptake by cancer cells in a mouse B16F10 melanoma model
Source: EJNMMI Res. 2018 Aug 16;8:82. doi: 10.1186/s13550-018-0433-1 (PMC6095935; doi:10.1186/s13550-018-0433-1)
Supplement: Supplementary file 1 — Figure S1. Flow-cytometry analysis of metabolism of glucose. a Flow-cytometry analysis of high expression cells of glucose metabolism markers; GLUT1 and hexokinase II. Values represent percentage of CD45− cancer cells (left) or CD45+ immune cells (right) in tumor on day 0 (n = 5). b Flow-cytometry analysis of high expression cells of glucose metabolism markers; GLUT1 and hexokinase II of CD45+ immune cells in spleen on day 0 (n = 5). Data represent mean ± SEM. Table S1. Complete data about flow-cytometry analysis of high expression cells of glucose metabolism markers; GLUT1 and hexokinase II of CD45+ immune cells in spleen on day 7. (PDF 194 kb) [file 13550_2018_433_MOESM1_ESM.pdf]

Anti PD-1 treatment increases [^{18}F]FDG uptake by cancer cells in a mouse B16F10 melanoma model

Supporting information

Mayu Tomita[1], Hironobu Yasui[2], Kei Higashikawa[2], Kohei Nakajima[1], Hideo Takakura[1], Tohru Shiga[3], Yuji Kuge[2], Mikako Ogawa[1]

[1]Graduate School of Pharmaceutical Sciences, Hokkaido University, Sapporo, Japan

[2]Central Institute of Isotope Science, Hokkaido University, Sapporo, Japan

[3]Graduate School of Medicine, Hokkaido University, Sapporo, Japan

Corresponding author: Mikako Ogawa, Laboratory of Bioanalysis and Molecular Imaging, Graduate School of Pharmaceutical Sciences, Hokkaido University, Sapporo, Hokkaido 060-0812, Japan.

Phone: +81-11-706-3767, Fax: +81-11-706-3767.

E-mail: mogawa@pharm.hokudai.ac.jp

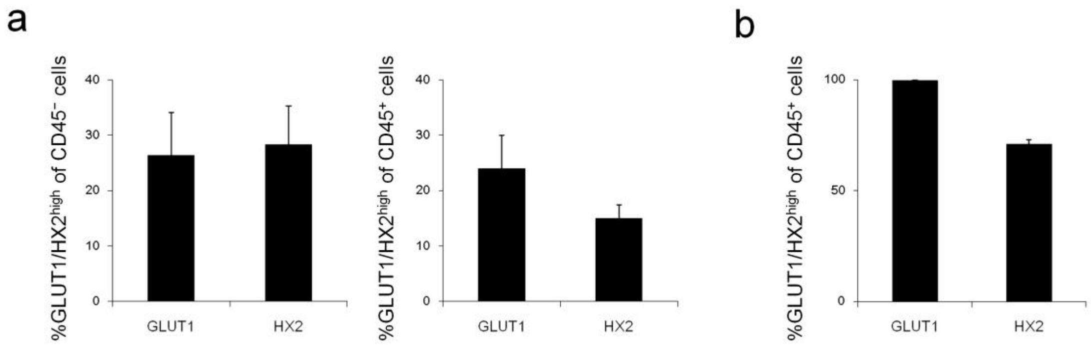

Fig. S1

Flow-cytometry analysis of metabolism of glucose. **a**, Flow-cytometry analysis of high expression cells of glucose metabolism markers; GLUT1 and hexokinase II. Values represent percentage of $CD45^{-}$ cancer cells (left) or $CD45^{+}$ immune cells (right) in tumor on day 0 (n = 5). **b**, Flow-cytometry analysis of high expression cells of glucose metabolism markers; GLUT1 and hexokinase II of $CD45^{+}$ immune cells ~~or~~ in spleen on day 0 (n = 5). Data represent mean ± SEM

Table S1

Complete data about flow-cytometry analysis of high expression cells of glucose metabolism markers; GLUT1 and hexokinase II of $CD45^+$ immune cells in spleen on day 7.

Group	Mouse	$\%GLUT1^{high}$ of $CD45^+$ cells	$\%HX2^{high}$ of $CD45^+$ cells
Treatment	mouse1	99.57	51.55
	mouse2	99.79	51.53
	mouse3	99.96	87.54
	mouse4	99.76	92.89
	mouse5	99.92	92.86
	mouse6	99.62	88.39
	Average	99.77	77.46
	SEM	0.06	8.25

Group	Mouse	$\%GLUT1^{high}$ of $CD45^+$ cells	$\%HX2^{high}$ of $CD45^+$ cells
Non-treatment	mouse7	99.66	53.40
	mouse8	99.29	55.35
	mouse9	99.74	88.43
	mouse10	99.72	95.62
	mouse11	99.26	91.79
	mouse12	99.44	88.76
	Average	99.52	78.89
	SEM	0.09	7.83
